# Supplementary material for: Extensive Angular Sampling Enables the Sensitive Localization of Macromolecules in Electron Tomograms
Source: Int J Mol Sci. 2023 Aug 29;24(17):13375. doi: 10.3390/ijms241713375 (PMC10487639; doi:10.3390/ijms241713375)
Supplement: Supplementary file 1 [file ijms-24-13375-s001.zip › ijms-2539579-supplementary.pdf]

Supplementary Figures

Extensive Angular Sampling Enables the Sensitive Localization of Macromolecules in Electron Tomograms

Marten L. Chaillet [1], Gijs van der Schot [1], Ilja Gubins [2,†], Sander Roet [1], Remco C. Veltkamp [2] and Friedrich Förster [1,*]

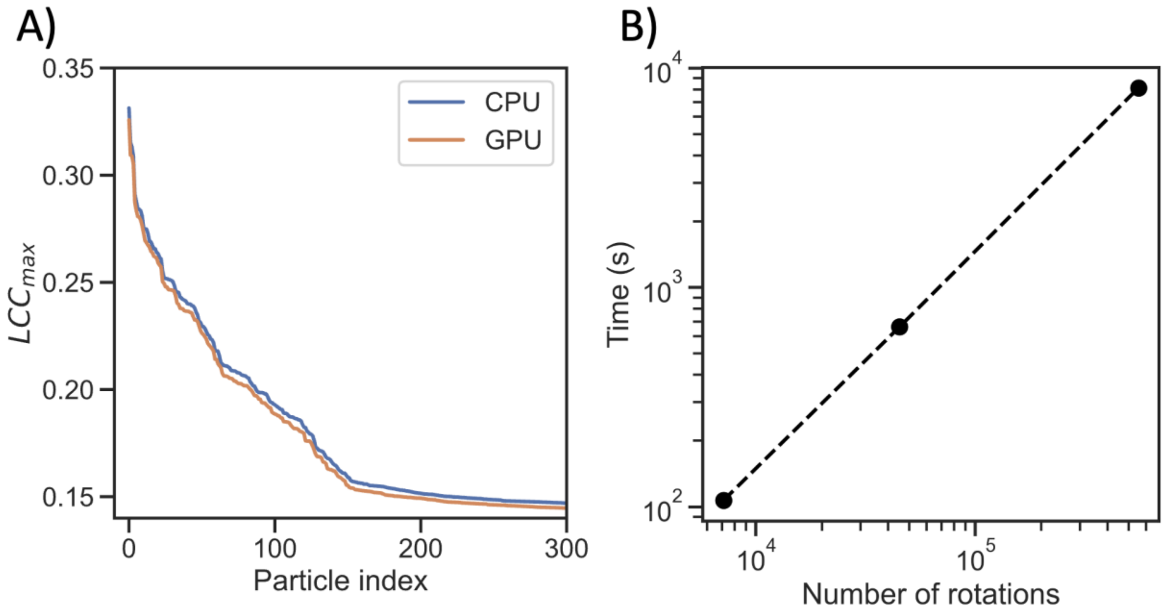

Figure S1. Assessment of GPU TM implementation. (A) LCC_{max} values ordered from high to low for the 300 highest ranking particles are consistent between the CPU (blue) and GPU (orange) implementation. (B) Compute time increases linearly with the number of rotations sampled with the same resources. The dotted line is a linear curve drawn between the points. Both time and the number of rotations are displayed on log scaled axes.

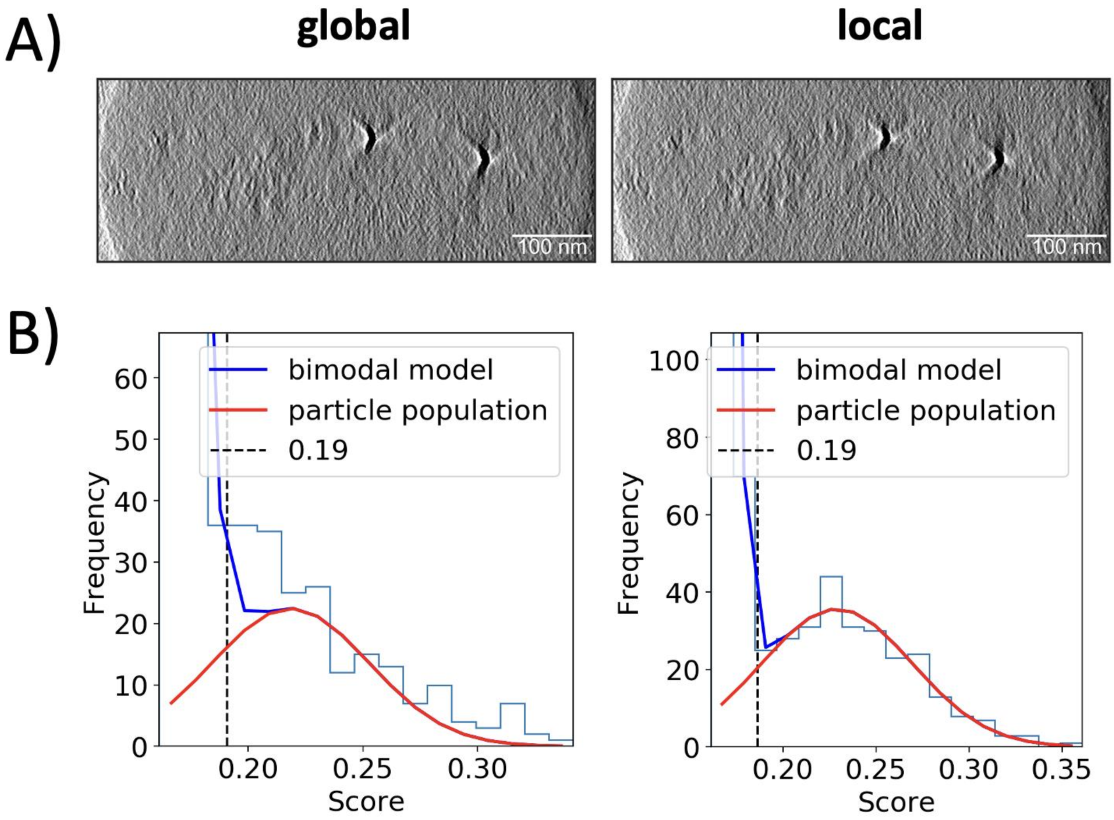

Figure S2. Automated particle classification improves when correcting for local motion. (A) Slice of the globally and locally aligned tomogram for the tilt-series with high fiducial residuals for the rigid body fit (Figure 3). The slice is along the y-axis (or xz-plane) shown between +/- 3σ. (B) Evaluation of performance of LCC_{max} as a classifier for globally and locally aligned tomograms. Histogram of the LCC_{max} values are shown with the Gaussian fitted to the true positives in the candidates (red line) and the bimodal model of the background and positives (blue line), the dashed line illustrates the LCCmax cutoff estimated from the RUC.

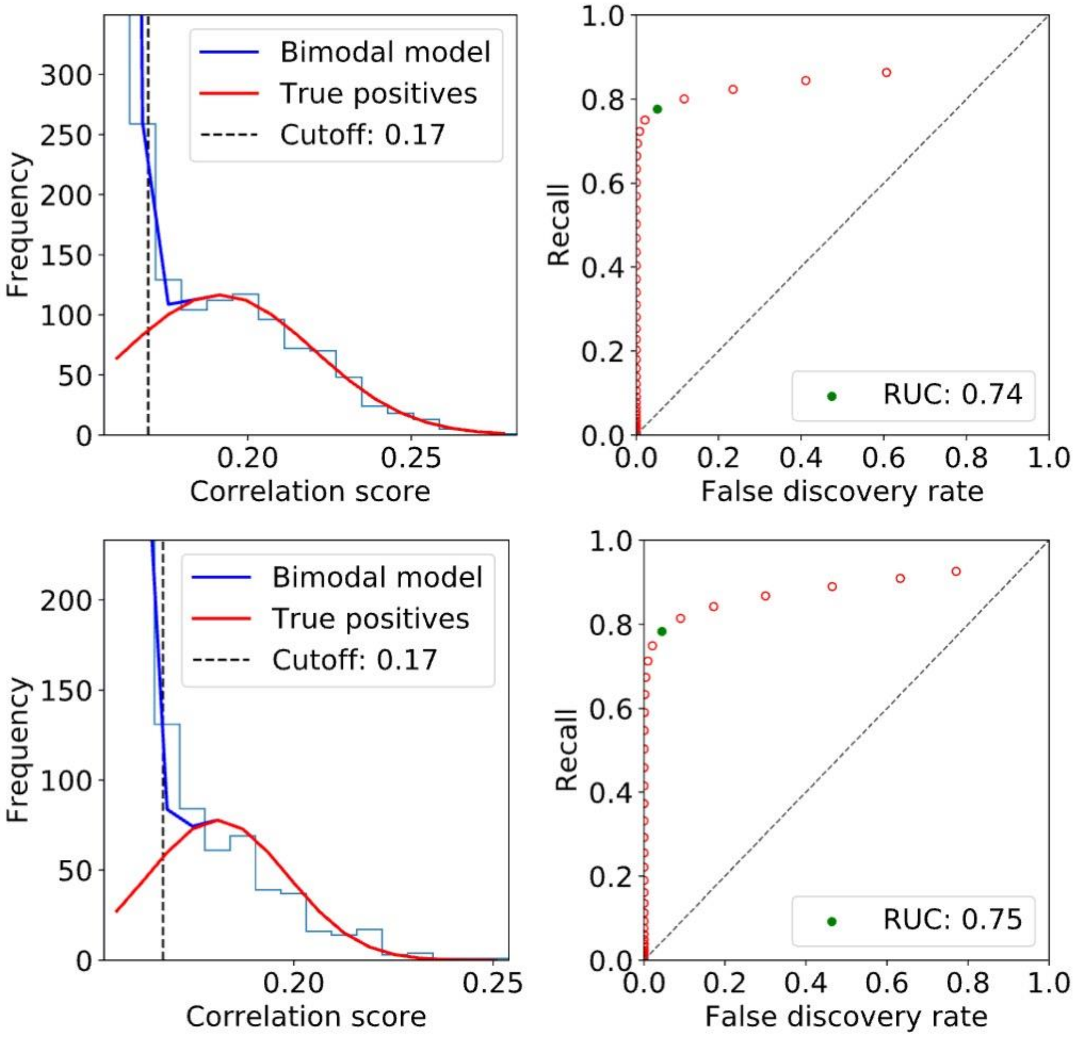

Figure S3. Histogram fit for plasma FIB lamellae. Evaluation of performance of LCC_{max} as classifier for two different plasma FIB-milled lamellae (top row corresponds to the left tomogram in Figure 4; bottom row corresponds to the right tomogram in Figure 4). Histogram of the LCC_{max} values on the left with the Gaussian fitted to the true positives in the candidates (red line) and the bimodal model of the background and positives (blue line), the dashed line illustrates the LCC_{max} cutoff estimated from the RUC. On the right the corresponding ROC-curves are shown (red dots) with the RUC point (green dot) that provides a good balance between recall and FDR.

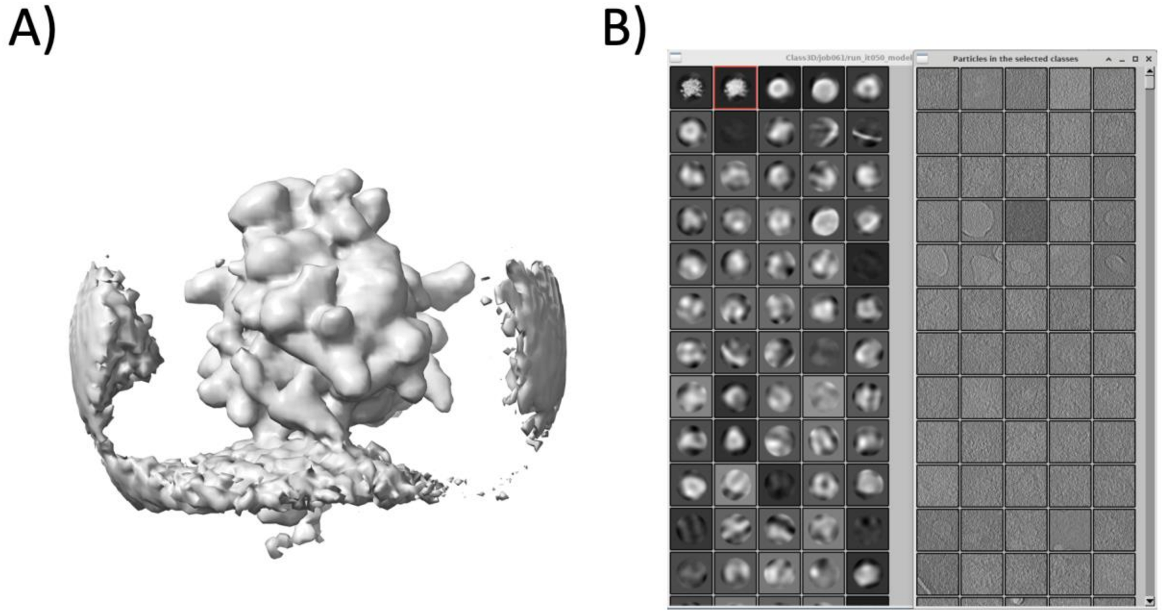

Figure S4. False positive removal in subtomogram classification. (A) Initial subtomogram average. (B) 3D classification to filter false positives. First 60 classes (of 100 total classes) are shown on the left, class 2 is highlighted in red and contained a mixture of ribosomes and ice artifacts. On the right a small group of the subtomograms of class 2 are shown which shows mostly ribosomes but also some ice blobs.
